# Meta-analysis of the effects of mental health literacy intervention on teachers: knowledge, stigma, help-seeking, and helping

**DOI:** 10.3389/fpsyg.2025.1700220

**Published:** 2026-01-07

**Authors:** Chunyu Liang, Xinyong Zhang, Yuxuan Chen

**Affiliations:** 1 Shapu Middle School, Guangzhou, Guangdong, China; 2 Department of Applied Psychology, Guangdong University of Foreign Studies, Guangzhou, Guangdong, China

**Keywords:** mental health literacy, teachers, pre-service teachers, intervention, meta-analysis

## Abstract

Given its critical role in promoting students’ mental health and wellbeing, research on mental health literacy has increasingly focused on both in-service and pre-service teachers. We conducted this meta-analysis to examine the effectiveness of mental health literacy interventions aimed at teachers (including pre-service teachers) in improving mental health knowledge, stigma, help-seeking behaviors, and helping behaviors, while also exploring the broader impact of these interventions. Studies were identified by searching five databases (PubMed, PsycARTICLES, ERIC, Web of Science, and the Cochrane Controlled Trials Register). Of the 6,186 references identified, 18 studies were included in the meta-analysis based on the selection criteria. The results were as follows: (1) Post-training effects ranged from small to large for knowledge (g = 1.08), stigma reduction (g = −0.33), and helping behaviors (g = 0.48). (2) At 3-month follow-up, only knowledge and helping showed significant medium-to-large effects (knowledge: *g* = 0.73, helping: *g* = 0.52). (3) At longer follow-ups (> 3 months), effects on knowledge and helping remained significant (knowledge: *g* = 0.53, helping: *g* = 0.55). (4) No significant improvements were observed in help-seeking behaviors. (5) Subgroup analyses showed no significant moderating effects of region, participant type, experimental design, or intervention interaction modality for knowledge. For stigma, region, participant type, and experimental design significantly moderated intervention effects, whereas no significant moderators were observed for helping behaviors. This study indicates that mental health literacy interventions for teachers effectively improve knowledge, reduce stigma, and enhance helping behaviors in the short term, but not in promoting help-seeking. Intervention effects on knowledge remain stable over time, while the effects on stigma and helping behaviors are less consistent. Future research should include longer follow-up periods to assess sustained effects and investigate additional moderators of intervention effectiveness.

## 1 Introduction

Intervention research on mental health literacy (MHL) encompasses diverse populations across the lifespan, from adolescents to older adults, including healthcare professionals, community members, students, and educators. Both intervention content and delivery methods have become increasingly diversified and standardized (Ming and Chen, 2020). Systematic reviews consistently demonstrate that most MHL interventions show efficacy in improving mental health literacy (Clement et al., 2015; Freţian et al., 2021; Maunder and White, 2019; Nazari et al., 2023). Given that awareness is a key driver for the receipt and provision of mental health services (Cai et al., 2023; Chen et al., 2023; Wang et al., 2025), improving teachers’ MHL is crucial for creating supportive school environments that can respond to students’ mental health needs.

The pivotal role of teachers in student mental health is well established. Globally, approximately one-fifth of the pediatric population experiences mental health challenges (Kieling et al., 2011), and educators are key in promoting wellbeing. The National Institute of Mental Health reported that 50% of individuals with a lifelong mental illness experience onset by middle school (Kuehn, 2005). As students spend most of their weekday hours in school, teachers are often the first to notice signs of mental health concerns and serve as primary contacts for students seeking guidance (Ma et al., 2022a, 2022b; Whitley et al., 2013).

MHL, originally conceptualized by Jorm et al. (1997) as “knowledge and beliefs about mental health problems which aid their recognition, management or prevention,” has undergone significant theoretical evolution. The concept was later expanded to include help-seeking, self-help, and helping others (Jorm, 2012), and further broadened by Canadian scholars to incorporate stigma reduction and mental health promotion (Kutcher et al., 2016). Consequently, MHL now encompasses four key dimensions: (a) knowledge acquisition and maintenance of positive mental wellbeing; (b) comprehension of mental disorders and their treatments; (c) reducing stigma associated with mental illness; and (d) enhancement of help-seeking behaviors and self-care competencies.

Despite the clear importance of MHL, a substantial body of evidence highlights critical gaps in teachers’ competencies. Studies across various nations reveal low correct recognition rates of mental disorders, particularly in developing countries—for instance, only 16.3% of Nigerian teachers correctly identified depression, and 44.2% of Pakistani teachers recognized autism as a mental disorder (Aluh et al., 2018; Ayub et al., 2017; Javed et al., 2006). Prevalent negative attitudes and high levels of stigma are also well documented, with cross-sectional surveys indicating that a significant proportion of teachers perceive individuals with mental illness as dangerous or unpredictable (Aluh et al., 2018; Fekih-Romdhane et al., 2022; Janoušková et al., 2017). Furthermore, teachers often exhibit negative attitudes toward seeking professional psychological help themselves, preferring informal support (Uzman and Telef, 2015), and report low confidence in their ability to assist students with mental health problems, indicating a need for additional professional development in this area (Chorcora and Swords, 2021; Fortier et al., 2017; Ueda et al., 2021).

In response to these identified needs, intervention research on MHL among teachers and pre-service teachers is increasing (Wei et al., 2021). Multiple standardized MHL training programs have been introduced, including modified versions for school environments in countries like Canada and the United States (Kutcher et al., 2013; Miller et al., 2019). For example, Australia’s Youth Mental Health First Aid (Y-MHFA) trains teachers in crisis intervention strategies, with studies showing significant knowledge gains retained at 6-month follow-up (Jorm et al., 2010; Sánchez et al., 2021). Non-standardized, short-term training—delivered through lectures, videos, group discussions, and online learning—also represents a common and relatively cost-effective intervention method, demonstrating effectiveness in improving knowledge and willingness to help (Matsuda, 2021; Powers et al., 2014; Ueda et al., 2021; Vieira et al., 2014).

Previous studies have demonstrated that teachers often exhibit low levels of MHL, reflected in limited knowledge and persistent stigma toward mental illness. Although existing interventions have shown promising improvements, the evidence base remains methodologically constrained (Fazel et al., 2014). Many studies employed small samples, relied on self-report instruments (often non-validated), used short follow-up periods, or were non-randomized in design. These limitations restrict the generalizability of findings and underscore the need for a comprehensive synthesis.

This review addresses these gaps by conducting a systematic review and meta-analysis of MHL interventions for teachers. Specifically, we quantify the effects of such interventions across four prespecified domains—knowledge, stigma, help-seeking, and helping—while examining both short-term and longer-term outcomes. In addition, we assess the robustness of findings through sensitivity analyses that account for study quality and outcome measure validity. By doing so, this review contributes novel evidence to inform the design of effective, sustainable MHL programs for educators and provides policy-relevant insights into how improving teacher MHL may ultimately benefit student wellbeing.

## 2 Methods

This review followed the Preferred Reporting Items for Systematic Reviews and Meta-Analyses (PRISMA) guidelines as established by Liberati et al. (2009). The protocol was prospectively registered with the International Prospective Register of Systematic Reviews (PROSPERO, www.Crd.york.ac.uk/prospero) on 17 December 2022, registration number: CRD42022380424 (amended version dated 5 January 2023).

### 2.1 Inclusion and exclusion criteria

Studies meeting the following eligibility criteria were incorporated into the analysis:(1) studies with interventions aimed at improving MHL; (2) population: schoolteachers (including pre-service teachers), mainly from primary/elementary, middle, secondary, or high school, but university or college teachers were also included. When teachers were included and their outcomes were presented separately, studies examining mental health literacy targeting community members were also included; (3) RCT and quasi-experimental (QE) designs; (4) outcomes: including at least one of the following: knowledge of mental illness and its treatment, stigma or attitude toward mental illness, help-seeking behavior, confidence in assisting students with mental health problems, intention to provide help or engage in helping behavior; (5) published as a journal article and or report; (6) no restriction on date of publication.

Exclusion criteria were: (1) studies that used secondary data to assess primary outcome measures or reviews; (2) studies without reported results; (3) qualitative studies; (4) studies not published in English.

These criteria were established to ensure the inclusion of studies that directly assessed teacher-focused MHL interventions with quantifiable outcomes, thereby enhancing comparability and minimizing confounding effects.

**Figure 1:**
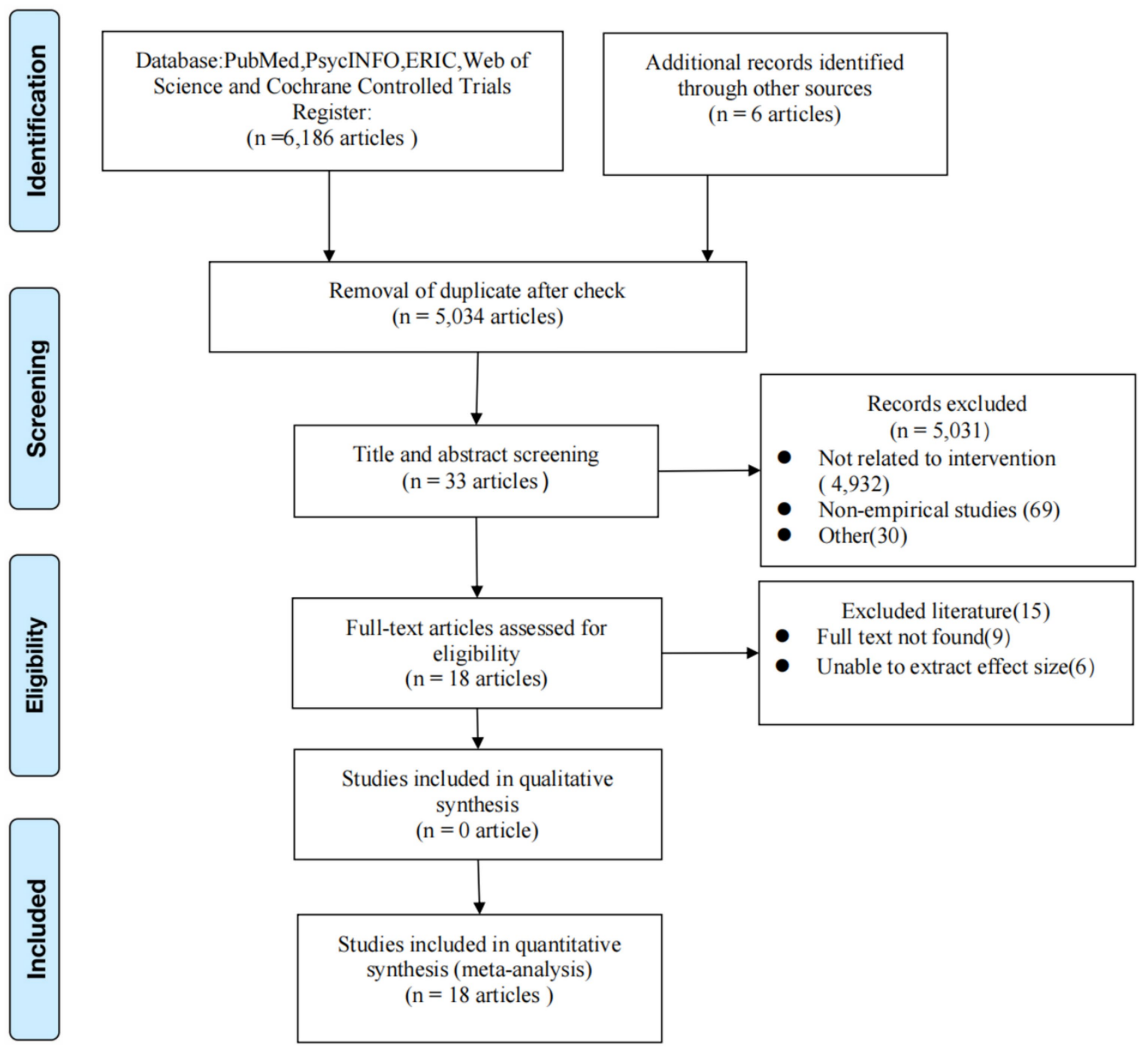
Flow chart of literature search and screening.

Out of a total of 6,186 studies identified, 18 met the eligibility criteria (Figure 1).

### 2.2 Search strategy and study selection

Five electronic bibliographic databases, including PubMed, PsycINFO, ERIC, Web of Science, and Cochrane Controlled Trials Register, were searched in November 2022. Search terms included “mental disorder,” “mental health,” “mental illness”; “seek care,” “seek help,” “help seek,” “help-seeking”; “knowledge,” “attitude”; “curriculum,” “health promotion,” “health education”; “teacher,” “educator,” and related terms.

The initial screening of titles and abstracts was performed independently by a reviewer (Liang), who removed irrelevant studies. A second reviewer (Zhang) then verified these exclusions. For the final study selection, two reviewers (Liang and Zhang) independently assessed the full-text articles for eligibility. Any discrepancies were resolved through consultation with a third reviewer (Hu).

### 2.3 Data extraction and quality assessment

Data were extracted in duplicate by two authors (Liang and Zhang) working independently. Any disagreements were adjudicated by an independent reviewer (Hu) to ensure consistent interpretation. The following information was extracted and coded from each study: author, year, country, study design, teacher type, measurement time points, sample size, participant age, method of recruitment, type of intervention, intervention topic, duration of intervention, measurement tools, measured outcome, and results of the quality assessment. According to previous reviews, four types of outcomes were extracted (Jiang et al., 2020; Ming and Chen, 2020; Jorm, 2012; Kutcher et al., 2016). Four outcome domains were harmonized across studies. Knowledge included validated or study-specific tests of mental health knowledge. Stigma encompassed measures of public stigma, personal stigma, or attitudes toward individuals with mental illness. Help-seeking referred to self-reported intentions or behaviors related to seeking help for one’s own mental health. Helping comprised helping intentions, confidence/self-efficacy, and reported helping behaviors directed toward students or peers.

Data extraction was conducted according to the following rules: (1) If the scale contained both total and subscale scores, the total score was extracted first. If only subscale scores were available, the subscale scores significantly related to the outcome were extracted (Ren et al., 2020); (2) if both attitude and stigma were measured in a study, the stigma score was extracted first; (3) if a study included helping behavior, helping intention and helping confidence, the scores for helping behavior were extracted first.

The National Institutes of Health (NIH) quality assessment tool was used to assess the scientific quality of the included studies.^1^ Randomized controlled studies used *the Checklist for Quality Assessment of Controlled Intervention Studies*, comprising 14 questions. Two raters scored each question separately, with 1 point awarded for each ‘yes’ response, excluding Item 13 (prespecified outcomes/subgroups). Studies with preregistration were awarded 1 point, those that declared outcome changes received 0.5 points, and those without registration received 0 points. Blinding measures (Item 2) were not included in the scoring system, as they are generally unfeasible in educational intervention research (Freţian et al., 2021). The final possible scores ranged from 0 (indicating very high risk of bias) to 13 (indicating very low risk of bias). *The Checklist for Quality Assessment Tool for Before-After (Pre–Post) Studies With No Control Group* was used for non-randomized studies, comprising 12 questions. Each question was scored separately by two authors, with 1 point assigned to each question answered “yes.” The final possible scores ranged from 0 (indicating very high risk of bias) to 12 (indicating very low risk of bias). To evaluate the impact of study quality, we prespecified sensitivity analyses that excluded studies rated as high risk of bias (score < 4).

Two independent assessors (Liang and Chen) conducted quality assessments, followed by calculation of inter-rater reliability. Inter-rater disagreements were adjudicated by an independent third reviewer (Zhang) to achieve consensus. When the final judgment differed between the two reviewers, they discussed with the third reviewer (Zhang) to reach a consensus. The Kappa coefficient was used to measure inter-rater reliability, which was 0.767 in this study. According to the criteria of 0.75 or above, the agreement was considered very good (Orwin et al., 1994), indicating high reliability of the quality assessment in this study.

### 2.4 Calculation of effect size

A comprehensive meta-analysis was conducted on four key outcome variables (mental health knowledge, stigma, help-seeking, and helping behaviors) using Hedges’ *g* as the effect-size metric, which incorporates a correction factor for small sample sizes relative to Cohen’s *d* (Ren et al., 2020). The analysis was conducted by one author using the Comprehensive Meta-Analysis (CMA) Version 3.3. Hedges’ *g* effect sizes for RCTs were computed using intervention and control group sample sizes along with post-test means and standard deviations. When these parameters were unavailable, conversion formulas employing *χ^2^*, *t*, or *F* statistics were applied for estimation. For pre–post studies lacking control groups, Hedges’ *g* effect sizes were computed using pre-intervention and post-intervention means, sample size, and paired *t* values to account for within-subject dependencies. Effect sizes were calculated using a random-effects model, and moderators were analyzed using mixed-effects models. Effect sizes were categorized as small (0.2–0.5), medium (0.5–0.8), and large (>0.8) (Liu et al., 2011).

### 2.5 Data analysis

Analyses were conducted using CMA Version 3.3, employing random-effects models with *Hedges’ g* as the standardized effect-size metric. Publication bias was assessed with funnel plots, the fail-safe number (*N_fs_*), and Egger’s test (Egger et al., 1997). First, the funnel plot and *N_fs_* were used for testing (Chen et al., 2019), and then further combined with the Egger linear regression method for testing (Ren et al., 2018; Odgers et al., 2020). Symmetrical, centrally distributed funnel plots generally indicate low likelihood of publication bias, whereas asymmetry may suggest potential publication bias or small-study effects (Sutton and Rothstein, 2005). A minimum of 10 studies is generally recommended for the reliable interpretation of funnel plots and Egger’s test (Morgan et al., 2018a, 2018b). A statistically significant Egger intercept (i.e., intercept significantly different from zero with *p* < 0.05) suggests the presence of small-study effects or potential publication bias. According to Rothstein et al. (2005), publication bias may be possible when the fail-safe N is less than 5n + 10, where n is the number of studies.

To evaluate the stability of our findings, we conducted sensitivity analyses by recalculating effect sizes after excluding statistical outliers, defined as studies whose 95% CI did not overlap with the 95% CI of the pooled effect size (Note: studies whose 95% CI included zero were considered null studies and were examined separately in sensitivity checks) (Morgan et al., 2018a, 2018b).

Heterogeneity was measured by Higgins’ *I^2^* and *Q* statistic. Low heterogeneity was indicated by a value of 0–50%, moderate heterogeneity by 50–75%, and high heterogeneity by 75–100% (Higgins, 2003). When *Q* is significant and *I^2^* ≥ 75%, it indicates non-negligible, high heterogeneity among studies. Further subgroup analyses were performed, where *I^2^* was not zero and the confidence interval encompassed high levels of heterogeneity, to investigate possible reasons for heterogeneity. To ensure the reliability of subgroup analyses and avoid misleading estimates from small numbers of studies, a minimum of four studies per subgroup was set as the cut-off, in line with methodological recommendations for meta-analysis (Higgins et al., 2019). Guided by evidence-based medicine principles and prior research findings, this study considered four PICO aspects—population, intervention, comparison, and outcome—to propose possible factors influencing MHL intervention effects: (1) region and type of teachers; (2) intervention type; (3) study design; (4) outcomes: knowledge, stigma, help-seeking, and helping behaviors.

## 3 Result

### 3.1 Study description and study quality

We identified 6,186 possible records from the literature search, of which 1,152 were duplicates. Additionally, six records were identified through manual searching (Figure 1). Finally, 18 articles met the inclusion criteria.

Table 1 summarizes the characteristics of the 18 included studies, comprising 12 from developed nations (5 from Canada, 3 from the United States, 2 from Australia, 1 from Spain, and 1 from Japan), 4 from developing countries (2 in Pakistan, 1 in Saudi Arabia, 1 in Nigeria), and 2 from least developed regions (1 in Tanzania, 1 in Malawi). The methodological designs included 1 cluster RCT, 5 RCTs, and 12 non-randomized pre–post studies, with 7 studies conducting follow-up assessments (6 within 3 months, 1 beyond 3 months). Participant demographics consisted of pre-service teachers (4 studies), primary school educators (4 studies), secondary school educators (6 studies), and mixed primary/secondary cohorts (4 studies). Sex representation was predominantly mixed across studies, with one study exclusively examining male participants (Alshehri et al., 2020). Using the NIH quality assessment tool to evaluate study quality, we found that the RCT group included one study with a score of 5, one study with a score of 6, two studies with a score of 7, one study with a score of 9, and one study with a score of 11. The average score was 7.5 (*SD* = 1.11). The non-RCT group included one study with a score of 4, four studies with a score of 5, four studies with a score of 6, two studies with a score of 7, and one study with a score of 8. The average score was 5.83 (*SD* = 2.17).

**Table 1:** Characteristics of included studies.

No.	Study	Country	Study design	Measuring tool	Way of recruitment	Teacher type	Sample size	Measurement time point	Participant age (mean)	Participant age range	Type of intervention (materials and procedures)	Intervention topic	Duration	Outcome	Assessment results of research quality
1	Jorm et al. (2010)	Australia (developed country)	Cluster RCT	Self-developed questionnaires and vignettes	Voluntary	Secondary	327 (IG and CG: 221，106)	Pre-test; post-test; and follow-up of 6 months	NR	NR	Education: the Youth Mental Health First Aid course	General MH	2 d (7 h × 2)	Knowledge Stigma Helping	9
2	Chaves et al. (2021)	Spain (developed country)	RCT	An *ad hoc* vignette-based questionnaire; the Attribution Questionnaire-9 (referring to the vignette); a questionnaire of general knowledge about OCD	Voluntary	Primary and secondary	95 (IG and CG: 49，46)	Pre and post	43.29	24–65	Education: paper materials(an educational fact sheet about OCD)	Specific MH: OCD	Brief intervention	Knowledge Stigma	5
3	Ueda et al. (2021)	Japan (developed country)	RCT	An assessment questionnaire combining self-developed items and items from existing measures (the Depression Stigma Scale (DSS) and Japanese version of the Reported and Intended Behavior Scale (RIBS-J))	NR	All types	92 (IG and CG: 49，43)	Pre and post	41.2	NR	Education: video (anime film)	General MH	50 min	Knowledge Stigma Helping	6
4	Imran (2022)	Pakistan (developing country)	RCT	A questionnaire developed by the WHO; a Likert scale to assess teachers’ confidence in helping students with mental health problem	Voluntary	Primary and secondary	231 (IG and CG: 118，113)	Pre-test; post-test; and follow-up of 3 months	32.6; 32.3	NR	Education: workshop (the teachers’ training curriculum based on WHO-EMRO School Mental Health Manual)	General MHL	3 d (3 × 6 h)	Knowledge Helping	11
5	Alshehri et al. (2020)	Saudi Arabia (developing country)	RCT	Self-developed questionnaires of ADHD knowledge	NR	Primary	100 (IG and CG: 50，50)	Pre-test; post-test; and follow-up of 3 months	NR	NR	Education: workshop; lecture; paper materials	Specific MH: ADHD	2 d	Knowledge	7
6	Greif Green et al. (2020)	United States (developed country)	RCT	Gatekeeper Behavior Scale; Teacher Mental Health Vignette Scale; the Reported and Intended Behavior Scale (RIBS)	Voluntary	Pre-service	46 (IG and CG: 24，22)	Pre-test; post-test; and follow-up of 1 month	NR	NR	Online role-play simulation	General MH	NR	Helping Stigma	7
7	Whitley et al. (2013)	United States (developed country)	Non-RCT	Self-developed 27-item, multiple-choice test of knowledge	Voluntary	Primary	Total pre-test: 134 intervention group and control group (pre–post): 52, 11	Pre and post	44	22–68	Education: workshop(PowerPoint slides)	Specific MH: OCD, ADHD, Tourette syndrome	2 h	Knowledge	5
8	Hussein et al. (2013)	Pakistan (developing country)	Non-RCT	Self-developed questionnaire of knowledge	Nominated	Primary	Pre: 140; post: 114	Pre and post	NR	NR	Education: interactive approach and a combination of video clips, handouts, and supporting materials	General MH	2 d(12 h)	Knowledge	6
9	Kutcher et al. (2013)	Canada (developed country)	Non-RCT	A 30-item self-developed questionnaire of knowledge about and attitudes toward mental health and mental disorders	Mandatory	Secondary	Knowledge—pre: 79; post: 79; attitude—pre:79; post:74	Pre and post	NR	NR	Education: Mental Health Curriculum Guide training program	General MH	1 d	Knowledge Stigma	6
10	Powers et al. (2014)	United States (developed country)	Non-RCT	A 27-item instrument developed by the research team at the participating university	Voluntary	Primary	150	Pre and post	NR	NR	Education: workshop (PowerPoint slides)	General MH	2 h	Knowledge	7
11	Kutcher et al. (2016)	Malawi (least developed country)	Non-RCT	An evaluation of mental health knowledge and attitudes was conducted using previously validated (Kutcher et al., 2013) written pre- and post-tests that were reviewed for cultural appropriateness by GCYDCA staff	NR	Primary and secondary	Knowledge—pre: 218; post: 218; attitude—pre: 218; post: 194	Pre and post	NR	NR	Education: workshop [based on the a school MHL curriculum African Guide: Malawi version (AGMv)]	General MH	3 d	Knowledge Stigma	6
12	Kutcher et al. (2016)	Tanzania (least developed country)	Non-RCT	An evaluation of mental health knowledge and attitudes was conducted using previously validated (Kutcher et al. 2013) written pre- and post-tests that were reviewed for cultural appropriateness by GCYDCA staff	Nominated	Secondary	Pre: 61; post: 38	Pre and post	NR	NR	Education: African Guide (AG) (a school MHL curriculum)	General MH	3 d	Knowledge Stigma	5
13	Carr et al. (2017)	Canada (developed country)	Non-RCT	Self-developed 30-item questionnaire of knowledge; an 8-item self-developed questionnaire to assess stigma; a 5-item self-developed questionnaire of helping intention and behavior	Voluntary	Pre-service	Knowledge—pre and post: 57; pre-test and follow-up: 39; Attitude—pre and post: 54; pre-test and follow-up: 34; Help-seeking—pre: 60; pre-test and follow-up: 38	Pre-test; post-test; and follow-up of 3 months	NR	NR	Education: professional development session based on a mental health literacy curriculum resource	General MH	1 d	Knowledge Stigma Help-seeking	5
14	Wei et al. (2021)	Canada (developed country)	Non-RCT	A 30-item knowledge questionnaire that was developed based on the content of the Guide resource; an 8-item questionnaire of stigma of mental illness; a 5-item questionnaire of attitudes about seeking help	Voluntary	Pre-service	Knowledge—pre and post: (43, 35); pre and follow-up: (13, 31) Stigma—pre and post: (49, 47); pre and follow-up: (15, 38); Help-seeking—pre and post: (48, 50); pre and follow-up: (14, 40)	Pre-test; post-test; and follow-up of 3 months	NR	NR	Education: online and face to face	General MH	2 d	Knowledge Stigma Help-seeking	5
15	Gilham et al. (2021)	Canada (developed country)	Non-RCT	The questions for knowledge, stigma, and help-seeking were developed by a psychiatrist and mental health researcher and reviewed by educators and other mental health professionals	Mandatory	Pre-service	Pre: 76; post: 71	Pre and post	NR	NR	Education: based on Bachelor of Education course (online modules and face-to-face class)	General MH	4 wk.(8–10 h)	Knowledge Stigma Help-seeking	8
16	Wei et al. (2021)	Canada (developed country)	Non-RCT	A 30-item questionnaire of knowledge; an 8-item questionnaire of stigma	Nominated	Secondary	Knowledge—pre and post: (949, 919); Stigma—pre and post: (949, 872)	Pre and post	NR	NR	Education: GETE(Go-To Educator Training) programs (using PowerPoint slides, video clips, group activities, and insession discussions)	General MH	2 d	Stigma Knowledge	7
17	Parker et al. (2021)	Australia (developed country)	Non-RCT	A 6-item adapted subscale of the Mental Health Knowledge Schedule (MAKS); a modified version of the Depression Stigma Scale—Personal Stigma subscale; an adapted version of the Confidence in Helping subscale; a 14-item adapted version of the Help Provided to Students questionnaire was used to measure the frequency of helping behaviors	Voluntary	Secondary	Pre: 70; post: 28; 3 mo: 23	Pre-test; post-test; and follow-up of 3 months	36.5	24–60	Education: a web-based training program(Building Educators’ skills in Adolescent Mental Health)	General MH	6 wk	Knowledge Stigma helping	6
18	Atilola et al. (2022)	Nigeria (developing country)	Non-RCT	Self-developed questionnaire assessed knowledge (8 items) and attitude (5 items) about depression, as well as their level of confidence (9 items)	Voluntary	Secondary	Pre: 294; post: 234	Pre and post	39.8	23–68	Education: an adapted version of the Break Free from Depression, a 4-module depression awareness curriculum	Specific MH:depression	NR	Knowledge Stigma Helping	4

NR, not reported; RCT, randomized control trial; CG, control group; IG, intervention group; Stigma: stigma or attitudes toward mental illness and attitudes regarding mental health; Knowledge: knowledge of mental illness (or specific mental illness); Helping: helping behavior and helping confidence; Help-seeking: mental health help-seeking behavior; MH: mental health; Nominated: nominated by principals or the Ministry of Education.

In general, most studies were carried out as educational interventions, with 17 studies using educational interventions and 1 study using online role-playing through an app. The intervention topics for most studies involved general mental health topics (*n* = 14). Some targeted specific mental health issues, namely OCD (*n* = 2), depression (*n* = 1), or ADHD (*n* = 1).

The included studies were conducted in educational contexts with varying student-to-teacher ratios and institutional resources. For instance, studies from least developed countries often reported larger class sizes and limited access to school-based mental health services, which may moderate intervention effectiveness. However, few studies provided explicit information on teacher workload or student-to-teacher ratios, limiting cross-country comparison.

Interventions varied in content and organization. Reported intervention durations ranged from less than 1 h to 12 h; however, nearly two-thirds of the studies (11 of 18) did not provide the exact duration. Among those that did, the shortest lasted 50 min. Most interventions were short term (≤3 days, *n* = 14). One was intermediate in length (up to 1 month), and one was long term (>3 months). Two studies did not report intervention duration. Seven studies adopted standardized MHL programs, including the Mental Health Curriculum Guide developed by Canadian scholars, the Adolescent Depression Awareness Program developed by the Johns Hopkins University School of Medicine in the United States, and the Mental Health First Aid (MHFA) program in Australia. Moreover, the means and forms of specific interventions are diverse, including educational lectures, group discussions, role-playing, videos, and online courses.

### 3.2 Effectiveness of interventions in improving MHL

This study analyzed intervention effects immediately post-intervention and at follow-up separately. The follow-up intervention assessments were temporally stratified into short-term follow-up (<3 months) and extended follow-up (≥3 months) intervals. Informed by extant literature on MHL moderators and our study cohort characteristics, we established the following analytic subgroups: (1) region: developed countries *versus* developing countries; (2) region: developed countries *versus* least developed countries; (3) teacher type: primary school teachers *versus* middle school teachers; (4) teacher type: pre-service teachers *versus* in-service teachers; (5) form of interaction between the interveners and participants: active interaction *versus* passive interaction; (6) study design: RCT *versus* non-RCT.

### 3.3 Evaluation of mental health knowledge

Among the 18 eligible studies, 17 assessed mental health knowledge outcomes. Sixteen studies (4 RCTs and 12 non-RCTs) were incorporated in the meta-analysis, as one study lacked extractable data for quantitative synthesis (Chaves et al., 2021). There are six follow-up studies. Of the studies included in the meta-analysis,15 studies reported significant post-intervention effects, and follow-up effects (1–6 months) were reported to be significant in five studies. A single study (Parker et al., 2021) failed to demonstrate statistically significant effects, with both immediate post-intervention and follow-up assessments showing null results.

Table 2 demonstrates substantial immediate post-intervention gains in mental health knowledge (*g* = 1.08), with high heterogeneity (*I^2^* = 97%). These improvements remained significant at the 3-month follow-up (*g* = 0.73) with high heterogeneity (*I^2^* = 77%), while effect sizes attenuated to moderate levels in longer-term assessments (*g* = 0.53).

**Table 2:** Intervention for mental health literacy: effect sizes, heterogeneity tests, and publication bias tests.

Outcome variables	*k*	*N*	*g*(95%CI)	Sensitivity test	Heterogeneity test			Publication bias test				
				*g*(95%CI)	*Q_W_*	*df*	*I^2^*	*Nf_s_*	Egger’s intercept	*SE*	95%CI	*p*
Knowledge												
Post-test	15	2,731	1.08***(0.78, 1.45)	1.14***(0.76, 1.52)	505.66***	14	97.23	3,948	−1.12	3.33	[−8.33， 0.08]	0.74
Within 3 months	4	398	0.73**(0.25, 1.12)	/	13.55**	3	77.87	/	/	/	/	/
After 3 months	1	327	0.53***(0.23, 0.77)	/	/	/	/	/	/	/	/	/
Stigma/attitude												
Post-test	11	2.072	−0.33***(−0.52, −0.15)	−0.64***(−1.01,-0.26)	90.642***	10	88.97	249	−1.66	1.53	[−5.11, 1.79]	0.31
Within 3 months	3	122	−0.24(−0.53, 0.05)	/	1.98	2	0	/	/	/	/	/
After 3 months	1	327	0.11(−0.16,-0.38)	/	/	/	/	/	/	/	/	/
Help-seeking												
Post-test	3	181	0.08(−0.52, 0.70)	/	21.35***	2	90.63	/	/	/	/	/
Within 3 months	1	54	0.15(−0.45, 0.76)	/	/	/	/	/	/	/	/	/
After 3 months	0	/	/	/	/	/	/	/	/	/	/	/
Helping												
Post-test	5		0.48***(0.21, 0.75)	0.63**(0.19, 1.06)	17.99***	4	77.76	67	2.49	1.94	[−3.69, 8.69]	0.29
Within 3 months	3		0.52***(0.29, 0.75)	/	2.22	2	9.55	/	/	/	/	/
After 3 months	1	327	0.55***(0.21, 0.89)	/	/	/	/	/	/	/	/	/

k represents the number of independent effect sizes, N is the sample size, and 95% CI is the 95% confidence interval for the effect size g corresponding to the outcome variable; **p < 0.01, ***p < 0.001. Heterogeneity test: Q_W_ represents the within-group heterogeneity test statistic (two-tailed test). Publication bias: 95% CI is the 95% confidence interval for the effect size g corresponding to Egger’s intercept (one-tailed test).

The results of the sensitivity analysis regarding mental health knowledge were as follows: 15 studies measured effects immediately after intervention. After removing one outlier (Parker et al., 2021), the effect size was *g* = 1.14 (*95%CI*: 0.76, 1.52), showing that the effects immediately post-intervention were robust. There were no outliers in the follow-up within 3 months and after 3 months. Therefore, the intervention effect of knowledge is robust.

Given the limited number of studies available for follow-up assessments (*n* < 10), we restricted our publication bias analysis to immediate post-intervention effects. The funnel plot demonstrates a symmetrical distribution of effect sizes, indicating minimal risk of publication bias for knowledge outcomes. *N_fs_* = 3,948 > 85, and the Egger linear regression intercept was not significant, *p* > 0.05 (*p* = 0.37). Therefore, the included studies show a low risk of publication bias (Table 2).

Subgroup analyses revealed no statistically significant moderating effects of geographical region, educator type, intervention delivery mode, or study design on mental health knowledge outcomes (Table 3).

**Table 3:** Results of subgroup analysis of knowledge.

Subgroup	k	*g*(95%CI)	Z	Q	I^2^(%)	X^2^	*p*
Region							
Developed country	9	1.20(0.68,1.71)	35.27***	283.27***	97.17	0.74	0.389
Developing country	4	0.86(0.29,1.43)	10.95***	67.18***	95.53		
Developed country	9	1.20(0.68,1.71)	35.27***	283.27***	97.18	0.22	0.637
Least developed country	2	1.06(0.79,1.32)	13.95***	1.92	47.89		
Type of the intervened							
Primary school teachers	4	0.79(0.66,0.97)	11.77***	1.069	0	0.00	0.984
Secondary school teachers	6	0.78(0.02,1.53)	29.27***	441.99***	98.87		
Pre-service teachers	13	1.96(0.99,2.92)	11.66***	8.12**	87.701	3.53	0.058
In-service teachers	2	0.95(0.56,1.34)	35.90***	477.65***	97.49		
Interaction between the interveners and the intervened							
Active interaction	11	1.143(0.69,1.59)	36.26***	470.02***	97.87	0.58	0.452
Passive interaction	4	0.893(0.42,1.37)	10.35***	18.79***	84.03		
Trial type							
RCT	11	0.87(0.70,1.03)	10.36***	48.15***	94.84	0.00	0.933
Non-RCT	4	1.12(1.06,1.19)	36.14***	439.25***	97.72		

95%CI is the confidence interval of 95% of the effect size g corresponding to the result variable; *p < 0.05, **p < 0.01, and ***p < 0.00.

Overall, MHL interventions produced moderate-to-large short-term improvements in teachers’ knowledge, but effect sizes tended to diminish at longer follow-up intervals. These findings suggest that knowledge gains are achievable but may require booster or sustained training to be maintained over time.

#### 3.3.1 Evaluation of stigma/attitudes

The meta-analysis incorporated 13 stigma/attitude studies (4 RCTs, 9 non-RCTs), with 6 showing immediate post-treatment effects and 2 exhibiting sustained effects during follow-up periods (1–6 months).

As shown in Table 2, mental health stigma/attitudes showed significant improvements at immediately after the intervention (*g* = −0.33), with high heterogeneity observed across studies (*I^2^* = 88%). No statistically significant differences were observed within the 3-month follow-up and after the 3-month follow-up.

The results of the sensitivity analysis regarding stigma/attitudes were as follows: After excluding six outlier studies (Jorm et al., 2010; Chaves et al., 2021; Parker et al., 2021; Ueda et al., 2021; Atilola et al., 2022) from the initial 11 post-intervention assessments, the adjusted effect size was g = −0.64 (95% CI: −1.01, −0.26). Two studies were included in the follow-up within 3 months, and one study was included in the follow-up after 3 months, all of which were outliers. Thus, the robustness of intervention effects on stigma/attitudes remains insufficient.

Owing to insufficient studies at follow-up time points (*n* < 10), only post-intervention effects were subjected to funnel plot analysis. Visual inspection revealed no evidence of publication bias for the stigma/attitude measures obtained directly after the intervention period. *N_fs_* = 249 > 65 and the Egger linear regression intercept was not significant, *p* > 0.05 (*p* = 0.153). Therefore, the included studies indicate a low risk of publication bias (Table 2).

Given the limited sample size, subgroup analyses were restricted to post-intervention stigma/attitude outcomes. These analyses revealed significantly greater intervention effects among educators in least developed countries compared with their counterparts in developed countries (*g*: −0.79 *vs*. −0.24, *p* < 0.001); the intervention effect for pre-service teachers was significantly better than that for in-service teachers (*g*: −0.95 *vs*. −0.27, *p* < 0.01); and the intervention effect in non-RCT studies was significantly better than in RCT studies (*g*: −0.42 *vs*. −0.04, *p* < 0.05) (Table 4).

**Table 4:** Results of subgroup analysis of stigma and attitudes.

Subgroup	k	*g*(95%CI)	Z	Q	I^2^(%)	X^2^	*p*
Region							
Developed country	8	−0.24(−0.42, −0.06)	−5.97***	28.97***	75.84	1.04	0.312
Developing country	1	−0.13(−0.25, 0.003)	−1.92	0	0		
Developed country	8	−0.24(−0.42, −0.06)	−5.97***	28.97	75.84	11.04	0.001***
Least developed country	2	−0.79(−0.94, −0.64)	−10.95	0	0		
Type of the intervened							
Primary school teachers	4	-	-	-	-		-
Secondary school teachers	6	-	-	-	-		
Pre-service teachers	1	−0.95(−1.37, −0.53)	−8.97***	79.41**	0	7.91	0.005**
In-service teachers	10	−0.27(−0.47, −0.10)	−4.44***	0	88.67		
Interaction between the interveners and the intervened							
Active interaction	3	−0.40(−3.51, −0.71)	−9.44***	88.97***	92.13	3.10	0.078
Passive interaction	8	−0.17(−0.34, −0.10)	−1.02	0.47	0		
Trial type							
RCT	3	−0.04(−0.23, 0.16)	−0.38	1.57	0	6.64	0.01*
Non-RCT	8	−0.42(−0.64,-0.2)	−9.83***	85.02***	91.77		

95%CI is the confidence interval of 95% of the effect size g corresponding to the result variable; *p < 0.05, **p < 0.01, and ***p < 0.001.

On average, interventions led to small-to-moderate reductions in stigma immediately post-intervention. However, high between-study heterogeneity and the limited number of studies in some subgroups mean that these effects should be interpreted with caution.

#### 3.3.2 Evaluation of help-seeking

Three non-randomized trials assessing help-seeking behaviors were included in the meta-analysis. Although two studies reported significant post-intervention improvements and Carr et al. (2017) documented significant follow-up changes, pooled analysis revealed no statistically significant immediate effects (*p* = 0.784, Table 2). The single available follow-up study similarly showed non-significant outcomes. In terms of follow-up effect within 3 months, the intervention effect of stigma/attitudes was also not significant. The included studies lacked follow-up after 3 months, so the intervention effect after 3 months was not analyzed. Evidence for changes in help-seeking was sparse, with only three studies contributing data. The pooled effect was non-significant, likely reflecting limited statistical power and short follow-up rather than a definitive absence of intervention effects.

#### 3.3.3 Evaluation of helping

The meta-analysis incorporated six helping behavior studies (4 RCTs and 2 non-RCTs) from the total of 18 included investigations. All six studies demonstrated statistically significant immediate post-intervention effects, with four including follow-up assessments. Among these, two maintained significant effects during 1–6 month follow-up periods.

Table 2 presents the helping behavior outcomes, demonstrating significant immediate post-intervention effects (*g* = 0.48) with substantial between-study heterogeneity (*I^2^* = 77%). These improvements persisted at 3-month follow-up (*g* = 0.52; *I^2^* = 9.5%), with effect sizes further increasing to moderate levels beyond this period (*g* = 0.55).

The results of the sensitivity analysis regarding helping were as follows: five studies measured effects immediately after the intervention. After removing two outliers (Jorm et al., 2010; Parker et al., 2021), the effect size was *g* = 0.63 (95%CI: 1.061, 0.192). There were no outliers in the follow-up within 3 months (3 studies included) and after 3 months (1 study included). Therefore, the intervention effect on helping is considered robust.

Owing to the limitation of the number of studies, only the subgroup analysis of post-intervention helping was carried out. Only the number of studies by region (developed countries *vs*. developing countries), form of interaction between the intervention providers and participants (active interaction *vs*. passive interaction), and study design (RCT *vs*. non-RCT) met the requirements for subgroup analyses. The results of the subgroup analysis were not significant (Table 5).

**Table 5:** Results of subgroup analysis of helping behaviors.

Subgroup	k	*g*(95%CI)	Z	Q	I2(%)	X^2^	*p*
Region							
Developed country	3	0.58(0.001, 1.15)	4.56***	13.40***	85.08	0.40	0.527
Developing country	2	0.38(0.15, 0.61)	5.55***	2.60	61.59		
Developed country	-	-	-	-	-		-
Least developed country	-	-	-	-	-		
Type of the intervened							
Primary school teachers	-	-	-	-	-		-
Secondary school teachers	-	-	-	-	-		
Pre-service teachers	-	-	-	-	-		-
In-service teachers	-	-	-	-	-		
Interaction between the interveners and the intervened							
Active interaction	3	0.35(0.22, 0.50)	5.89***	2.60	23.21	0.51	0.484
Passive interaction	2	0.71(−0.27, 1.70)	4.33***	11.68***	91.44		
Trial type							
RCT	3	0.67(0.22, 1.11)	6.18	10.22**	80.43	2.71	0.096
Non-RCT	2	0.78(0.15, 0.41)	4.37	0.14	0		

95%CI is the confidence interval of 95% of the effect size g corresponding to the result variable; *p < 0.05, **p < 0.01, and ***p < 0.001.

MHL interventions appear to increase teachers’ intentions, confidence, or behaviors related to helping others, although pooled estimates varied across studies. Given the small number of available trials and heterogeneity in measurement, these results should be considered preliminary.

## 4 Discussion

This study represents the first comprehensive meta-analysis to systematically evaluate the efficacy of MHL interventions among both in-service and pre-service teachers across primary and secondary education settings. The analysis examined how current interventions influence four key domains: knowledge, stigma/attitudes, helping behaviors, and help-seeking. Significant improvements were observed in knowledge, stigma/attitudes, and helping behaviors, but not in help-seeking. The review incorporated 18 studies, including RCTs and quasi-experimental designs. Although numerous studies have investigated knowledge and stigma/attitudes, fewer have examined help-seeking and helping behaviors. Geographically, most research has been conducted in developed countries, with studies on teachers’ MHL in developing and less developed countries remaining scarce and preliminary. Several methodological limitations emerged: (1) a paucity of high-quality RCTs, (2) most studies lacked follow-up assessments, limiting evidence for long-term effects, and (3) interventions were typically brief, one-time implementations with relatively short durations.

Regarding knowledge, the analysis revealed that both immediate post-intervention effects and maintenance effects remained significant, demonstrating moderate-to-large effect sizes. Regarding intervention effects, knowledge showed the most substantial improvement compared with stigma, help-seeking, and helping behaviors. Furthermore, the maintenance effect for knowledge was superior to that observed for stigma and helping behaviors. The results demonstrated that while the effect size diminished over time, it remained statistically significant at the 3-month follow-up and beyond, maintaining moderate-to-large effect sizes. These findings align with previous meta-analyses (Morgan et al., 2018a, 2018b; Amado-Rodríguez et al., 2022). These findings suggest that knowledge retention may decline over time without reinforcement. Future interventions should incorporate continuous learning components, such as supplementary online learning websites, videos, books, or other educational materials, to enhance knowledge consolidation.

The overall results indicated significant small improvements in stigma and attitudes immediately post-intervention. However, no significant improvements were observed at the 3-month follow-up or beyond, which is consistent with previous studies based on primary and secondary school settings (Amado-Rodríguez et al., 2022). However, this result differs from previous reviews of stigma and attitudes conducted in broader settings, where the follow-up effects were significant, showing effect sizes between small and medium (Freţian et al., 2021; Maunder and White, 2019). The observed discrepancies may stem from the exclusive focus on educational interventions implemented within primary and secondary school environments across all included studies. A previous review has shown that the most common approach to reducing stigma currently is education and contact (Morgan et al., 2018a, 2018b). Both face-to-face and video contact can effectively improve stigma and attitudes (Heim et al., 2019), but most current interventions for teachers or pre-service teachers use education only, and contact has not been applied to teachers. Given these findings, future studies should focus on investigating long-term outcomes (≥1 year) of MHL interventions. Additionally, the role of reinforcement strategies, such as booster training or continuous online learning modules, should be explored to maintain the gains in mental health knowledge and behaviors over time. These reinforcement strategies could potentially help mitigate the attenuation of intervention effects observed after the 3-month follow-up.

The meta-analysis found no statistically significant improvements in help-seeking post-intervention, differing from some previous meta-analytic conclusions (Gulliver et al., 2012; Ren et al., 2020). Current studies show no consistent evidence that MHL interventions improve help-seeking behavior (Brijnath et al., 2016). This discrepancy may arise because different MHL components variably affect help-seeking intentions across groups (Smith and Shochet, 2011). However, previous studies provided limited support for the efficacy of school-based MHL programs in enhancing help-seeking outcomes, such as attitudes, intentions, confidence, and actual help-seeking behaviors. Although these programs effectively enhance knowledge and reduce stigma, their translation into tangible help-seeking behavior remains inconsistent (Ma et al., 2022a, 2022b). This could be due to several factors, including high workloads of teachers, limited institutional support for mental health concerns, and teachers’ reluctance to seek professional help. Within the analyzed literature, only three studies incorporated help-seeking as an outcome variable, with varying quality of measurement tools, resulting in less conclusive evidence. Notably, all studies that included help-seeking as an outcome variable focused exclusively on pre-service teachers. This shows that current research has paid limited attention to help-seeking attitudes and behaviors among in-service teachers. This gap may reflect the predominant research focus on teachers’ mental health literacy as it relates to helping students rather than seeking help themselves.

For helping behaviors, immediate post-intervention effects were significant, with small-to-moderate and long-term follow-up effects remaining significant without decline, even showing an upward trend. This encouraging result may reflect that student-helping behaviors are inherently prosocial (Pfattheicher et al., 2022), and individuals with better MHL exhibit stronger prosocial attitudes and behaviors (Carvalho et al., 2022). However, previous reviews have not systematically evaluated MHL programs’ effects on helping behaviors, and existing studies rarely include helping as an outcome measure. Thus, we can only hypothesize that improved teacher mental health literacy during intervention enhances their self-efficacy in assisting students with psychological issues, thereby increasing helping behaviors. In the process of helping students, teachers may experience an increase in wellbeing and self-efficacy, and there is a mutually reinforcing relationship between prosocial actions and wellbeing (Bryant and Hui, 2022), which may lead to the maintenance of the effect on helping. Nevertheless, additional high-quality studies are required to conclusively determine the impact of mental health literacy programs on improving helping behaviors.

Through subgroup analysis, this study further examined how intervention outcomes (knowledge, stigma/attitudes, and helping behaviors) vary by region, teacher type, delivery format, and study design. Our analysis reveals significant regional disparities in stigma/attitudes improvement, with interventions demonstrating substantially greater effectiveness among teachers in less developed countries compared with their counterparts in developed nations. The least developed countries (LDCs) analyzed in this study—Malawi and Tanzania—are both African Nations. Cultural, socioeconomic, and religious factors in LDCs may reinforce more entrenched stigmatizing attitudes (Javed et al., 2021). In many LDCs, cultural beliefs and religious teachings often contribute to the stigmatization of mental illness. However, as MHL interventions challenge these beliefs by promoting a better understanding of mental health, the reduction of stigma may be more pronounced in these settings. However, these subgroup analyses are based on a small number of studies and require further research to draw definitive conclusions. Although this study focused only on socioeconomic factors, future research should systematically examine how cultural and religious contexts shape stigma in underdeveloped regions.

The changes of stigma and attitude are also different between pre-service teachers and in-service teachers, with more pronounced improvements observed among pre-service teachers. Regarding knowledge acquisition, the analysis revealed comparable intervention effects between pre-service and in-service teachers. This finding suggests that implementing MHL education during teacher training may represent a more effective approach for stigma reduction. However, randomized controlled trials are needed in the future to verify whether pre-service teachers indeed demonstrate superior improvements in stigma and attitudes.

The pre-service teachers included in the analysis were all students in Schools of Education, with most receiving blended (online and offline) instruction. The purpose of developing MHL education among pre-service teachers is to equip them with essential mental health competencies, thereby better preparing future educators for classroom demands (Gilham et al., 2021; Carr et al., 2017). This study suggests that both active and passive learning formats can be effective in improving teachers’ MHL. However, there is a need to examine the cost-effectiveness of different delivery methods, particularly in large-scale implementations. It is difficult to deliver MHL education to large numbers of in-service teachers due to various barriers in countries where social resources and human resources are scarce (Agyapong et al., 2023). However, standardized, curriculum-integrated interventions for pre-service teachers during on-campus training may prove more effective. On the one hand, the intervention period will be longer than that of short-term refresher training programs, which are more likely to yield sustained and meaningful improvements. On the other hand, such prolonged training ensures that pre-service teachers acquire and internalize the essential competencies for identifying and managing psychological distress in educational environments before entering the profession. Furthermore, the acquired knowledge and skills are reinforced and consolidated during the internship.

Although this study demonstrates that MHL interventions effectively enhance teachers ‘knowledge reserves and helping behaviors, it remains inconclusive whether these improvements directly translate into improvements in students’ mental health. Establishing such a causal chain presents multiple challenges: First, the improvement in teachers’ MHL (such as symptom recognition, stigma reduction, and enhanced confidence in helping) acts as an intermediate variable. Its impact on students must be transmitted through subsequent classroom practices, teacher–student interactions, and early identification/referral processes, which require more complex research designs for verification. Second, students’ mental health is influenced by multiple systemic factors, including family, peer relationships, community, and individual traits, making it difficult to assess teachers’ MHL contributions in isolation. Additionally, existing studies generally lack longitudinal data linking changes in teachers’ MHL with student mental health outcomes (e.g., symptom reduction, increased wellbeing, and greater help-seeking behaviors). Therefore, current evidence primarily supports the effectiveness of teachers’ MHL interventions at the teacher level. To confirm their downstream benefits on students’ mental health, future research should adopt cross-level longitudinal designs that measure teachers’ MHL, teacher behaviors, and students’ mental health outcomes, while employing path analysis or multilevel models to examine mediating pathways and causal mechanisms.

In conclusion, MHL interventions appear effective in enhancing teachers’ knowledge and reducing stigma in the short term, but evidence for sustained effects and behavioral change remains limited. Policymakers and curriculum designers should consider implementing sustained, contact-based programs that incorporate skills practice and longer follow-up periods to translate knowledge gains into lasting behavioral outcomes. Given teachers’ pivotal role in students’ daily lives, strengthening their MHL has the potential to improve not only educators’ own wellbeing but also the early identification and support of students with mental health needs. Future research should employ validated outcome measures, larger samples, and rigorous designs to consolidate the evidence base.

## 5 Limitation

This systematic review has several methodological limitations that warrant consideration. First, significant heterogeneity was observed across the included studies, potentially affecting the generalizability of the findings. Second, the overall methodological quality of the studies was suboptimal, with only six RCTs. Using a combined approach of quality assessment and sensitivity analysis, we evaluated study quality and identified several concerns: (1) There were 11 studies with an NIH quality assessment tool score below 7. In the RCT group, the main source of deviation was from outcome measurement. In the non-RCT group, the main sources of deviation were also from outcome measurement, and the detailed process of sample selection was not reported. (2) Despite these quality issues, sensitivity analyses confirmed the robustness of the primary findings regarding knowledge improvement and stigma/attitude changes. Although these limitations necessitate cautious interpretation, the core conclusions of this meta-analysis remain substantiated by the available evidence.

Other limitations include the use of various measurement tools for the same variables across studies, which complicates direct comparisons. Moreover, half of the studies used self-developed, unvalidated tools for measurement. Additionally, the small number of included studies further limits the analysis. Sensitivity analysis was only performed on the post-intervention effects of three outcome variables (knowledge, stigma/attitude, and helping), and not on help-seeking. Therefore, the robustness of the post-intervention effects could not be fully tested. Similarly, the robustness of the long-term effects of these four outcome variables could not be evaluated due to insufficient data. The statistical power for conducting comprehensive subgroup comparisons proved inadequate due to insufficient sample sizes across critical demographic strata. Regarding publication bias, analysis could not be conducted for the two outcome variables of help-seeking and helping, as the number of studies was less than 10. Furthermore, the classification of the four outcome variables was not sufficiently refined. For example, stigma was not differentiated into self-stigma and public stigma. Additionally, confidence in supporting students with psychological concerns, willingness to help, and actual helping behavior were all uniformly categorized as the helping outcome variable. Despite significant findings regarding the post-intervention and follow-up effects on knowledge, stigma/attitudes, and helping, the longest follow-up period among the included studies was only 6 months, and there was only one study with such a follow-up. Consequently, the current evidence base is inadequate for evaluating the sustained efficacy of these interventions. In terms of subgroup analysis, a limitation is that some subgroups are included in fewer studies, which may affect the stability of subgroup analysis results.

Our results were derived exclusively from peer-reviewed literature retrieved through established scientific databases, potentially omitting valuable data from alternative sources such as institutional reports, non-profit organization records, and community-based service documentation. Furthermore, the search strategy employed systematic terminology; certain relevant studies may have been inadvertently excluded due to limitations in keyword selection or database coverage constraints, suggesting the possibility of publication bias in our source materials.

Although scholarly interest in teachers’ MHL continues to grow, the current evidence base remains limited by a paucity of RCTs, which represent the highest standard for evaluating intervention efficacy (Yamaguchi et al., 2018). For future research, more randomized controlled experimental studies should be conducted, or the quality of non-randomized design studies should be improved to establish an empirical foundation for the effectiveness of teachers’ MHL interventions. Furthermore, it is essential to enhance the reporting methods and data quality in future studies. This can be achieved by providing more detailed information, such as the type of personnel responsible for delivering the intervention, the duration of the intervention, and the specific implementation methods used. The intervention period of most studies is relatively short. If it is difficult to extend the intervention period in future training programs, a variety of intervention methods may be considered, such as group discussions, film screenings, and contact-based activities. In addition, future research on the development of teacher MHL interventions based on school mental health curricula should focus on long-term outcome assessment, including follow-up measures of intervention effectiveness. To ascertain whether a single executed intervention is adequate or whether the goal should be to repeat treatments frequently, longer follow-up periods must be used to measure how long improvements are sustained.

## 6 Conclusion

The findings of our meta-analysis reveal that interventions are generally effective in enhancing MHL outcomes, including knowledge, stigma/attitudes, and helping behaviors in the short term, with the notable exception of help-seeking. However, over the long term, only improvements in knowledge are sustained. Our analysis further indicates that the effects on stigma/attitudes and helping behaviors are moderated by several factors, including region, teacher type, and study design. Notably, there is a significant gap in the literature regarding the limited number of randomized controlled trials and follow-up studies extending beyond 6 months. Future research should extend follow-up periods to better determine the long-term effectiveness of MHL interventions and further explore the factors that may influence the outcomes of these interventions.

## Data Availability

The original contributions presented in the study are included in the article/supplementary material, further inquiries can be directed to the corresponding author.

## Footnotes

^1^ https://www.nhlbi.nih.gov/health-topics/study-quality-assessment-tools
